# A link between premenopausal iron deficiency and breast cancer malignancy

**DOI:** 10.1186/1471-2407-13-307

**Published:** 2013-06-24

**Authors:** Jinlong Jian, Qing Yang, Yongzhao Shao, Deborah Axelrod, Julia Smith, Baljit Singh, Stephanie Krauter, Luis Chiriboga, Zhaoxu Yang, Jinqing Li, Xi Huang

**Affiliations:** 1 Department of Environmental Medicine, New York University (NYU), 550 First Avenue, New York, NY 10016, USA; 2 Department of Surgery, New York University (NYU), 550 First Avenue, New York, NY 10016, USA; 3 Department of Medicine, New York University (NYU), 550 First Avenue, New York, NY 10016, USA; 4 Department of Pathology, New York University (NYU), 550 First Avenue, New York, NY 10016, USA; 5 Department of Environmental Medicine, NYU Cancer Institute, NYU School of Medicine 550 First Avenue, New York, NY 10016, USA; 6 Xijing Hospital, Fourth Military Medical University, Xi’an 710032, P. R.China; 7 Tangdu Hospital, Fourth Military Medical University, Xi’an 710032, P. R.China

**Keywords:** Iron deficiency, Young breast cancer, Metastasis, Anemia, Epithelial mesenchymal transition, Hypoxia-inducible factors

## Abstract

**Background:**

Young breast cancer (BC) patients less than 45 years old are at higher risk of dying from the disease when compared to their older counterparts. However, specific risk factors leading to this poorer outcome have not been identified.

**Methods:**

One candidate is iron deficiency, as this is common in young women and a clinical feature of young age. In the present study, we used immuno-competent and immuno-deficient mouse xenograft models as well as hemoglobin as a marker of iron status in young BC patients to demonstrate whether host iron deficiency plays a pro-metastatic role.

**Results:**

We showed that mice fed an iron-deficient diet had significantly higher tumor volumes and lung metastasis compared to those fed normal iron diets. Iron deficiency mainly altered Notch but not TGF-β and Wnt signaling in the primary tumor, leading to the activation of epithelial mesenchymal transition (EMT). This was revealed by increased expression of Snai1 and decreased expression of E-cadherin. Importantly, correcting iron deficiency by iron therapy reduced primary tumor volume, lung metastasis, and reversed EMT markers in mice. Furthermore, we found that mild iron deficiency was significantly associated with lymph node invasion in young BC patients (p<0.002).

**Conclusions:**

Together, our finding indicates that host iron deficiency could be a contributor of poor prognosis in young BC patients.

## Background

Breast cancer (BC) patients diagnosed at < 45 years old have lower survival and higher recurrence rates when compared to their older counterparts. Cancers in these young patients are more likely to be of a higher histological grade and more readily metastasize to other organs [1,2]. The specific risk factors contributing to this poorer outcome have not been identified. Although a decline in total BC cases was recently reported, a plot of age-specific BC rates shows a decrease only in older women (≥ 45 years old), [3] suggesting that recent advances in BC research have just benefited older patients [4]. There is an urgent need to study young BC because despite poor prognosis in young patients, treatment options are the same as they are for older patients. This dilemma calls for new research to identify poor prognostic factors that specially belong to young women.

The differences in clinical outcomes between young and older patients cannot be fully explained by estrogen status and/or family history [5,6]. Studies in BC risk factors and tumor characteristics indicate that young BC patients have an additional disease entity that is unique to them [2,7]. A typical characteristic of young premenopausal women is the menstrual cycle. Because of menstruation, iron deficiency is highly prevalent in young women, particularly in poorer, less educated, and minority populations [8,9]. Interestingly, these populations also suffer more aggressive forms of BC than others [10]. This suggests that iron deficiency may be a risk factor in BC agressiveness of young patients, but its role has not been investigated to date.

The well-established “seed and soil” theory indicates that host responses are equally important as intrinsic properties of cancer cells in determining cancer outcomes [11]. For example, increasing evidence has demonstrated that iron has a role in the tumor microenvironment and pathways of iron acquisition, efflux, and regulation are all perturbed in cancer [12]. This suggests that reprograming of iron metabolism, either through iron responsive element/iron regulatory proteins or hepcidin/ferroportin axis or some unidentified mechanisms, is a central aspect of tumor cell survival [12] In distinction to increased iron in cancer cells and its microenvironment, we previously hypothesized that host iron deficiency and macroenvironment as a young women-specific risk factor results in increased susceptibility of young patients to BC metastasis and recurrence [13] Here we expand on this study and show that host iron deficiency promotes mammary cancer growth and metastasis in mice and is a significant predictor of lymph node invasion in humans.

## Methods

### Cell line and reagents

To avoid confounding factors such as estrogen, ER, PR, and Her2 negative human BC MDA-MB-231 and mouse mammary cancer 4T1 cell lines (ATCC, Manassas, VA) were used. Antibodies were: E-cadherin and Twist1 (Santa Cruz Biotechnology, Santa Cruz, CA); α-tubulin and Snai1 (Cell Signaling, Danvers, MA); Snai1 for immunofluorescence (Abcam, Cambridge, MA); Notch2 (Sigma Chemical Co., St. Louis, MO); and Alexa-488 labeled donkey anti-rabbit antibody (Invitrogen, Carlsbad, CA).

### Iron deficient mouse tumor xenograft models

All animal experiments were performed according to the protocol approved by the Institutional Animal Care and Use Committee at the New York University School of Medicine. The key to successfully induce iron deficiency in mice is to start the special diet at early age [14]. In the present study, 3-week-old immune-competent Balb/c mice (Jackson Laboratory, Bar Harbor, ME) were used. After randomly dividing into three groups, thirty mice were fed diets containing 3.5 ppm, 35 ppm, or 350 ppm ferrous sulfate, respectively. The diet was based on a commercial iron deficient AIN-93G purified rodent diet, which was found to contain 2 mg Fe per kg diet (2 ppm) [15]. The diets with different concentrations of ferrous sulfate were prepared by Dyets Inc. (Bethlehem, PA) and shipped to the laboratory. Upon receipt, iron concentrations and its availability were analyzed by ferrozine assay as previously described [16] and they all met the required specifications.

After 12 weeks, mice from each diet group were subcutaneously (*s*.*c*.) inoculated with 5 × 10^4^ 4T1 cells into the flanks (suspended in 0.1 ml PBS). Tumor volumes were measured twice weekly. Mice were sacrificed four weeks later and primary tumors, lung, and liver were collected. Each primary tumor was divided into three parts for RNA isolation, protein extraction, and histology, respectively. The upper lobe of the left lung was fixed in Bouin’s solution and lung metastases were quantitated by counting the numbers of tumor nodules on the surface.

To verify whether iron deficiency promotes tumor growth in human BC cells, twenty 3-week-old immuno-compromised non-obese diabetic/severe combined immune-deficient (NOD/SCID) mice were fed a special gamma irradiation-treated diet containing either 3.5 ppm or 350 ppm iron (Dyets Inc., Bethlehem, PA) for 12 weeks. After inoculating 1 × 10^6^ MDA-MB-231 cells into the mammary fat pads, tumor volumes were measured as described for 4T1 cells.

### RNA isolation and quantitative PCR (qPCR)

RNA from primary tumors was extracted in TRIzol (Invitrogen, Carlsbad, CA) and 10 μg RNA from each homogenate were pooled to represent the average expressions of the group. 2 μg pooled RNA were reverse-transcribed by Superscript® III RT (Invitrogen, Carlsbad, CA) [17]. The sequences of the primers are listed in Additional file 1: Table S1 and the mRNA expression levels of the target genes were normalized to the geomean of three housekeeping genes, GAPDH, G6PD, and HPRT1 and then expressed as fold changes over the mice fed 350 ppm iron diet.

### Protein extraction and Western blot

Primary tumor tissues were homogenized in RIPA lysis buffer containing proteinase inhibitors. 100 μg proteins were taken from each mouse and combined into one sample as representative of each group. Equal amounts of the pooled proteins were loaded onto 8-12% SDS-polyacrylamide gels. After transferring, PVDF membranes were first probed with primary antibodies, followed by HRP-labeled secondary antibody, and visualized by enhanced chemiluminescence kit (PerkinElmer, Waltham, MA).

### Immunofluorescence staining and immunohistochemistry

Fixed tumor tissues embedded in paraffin were sequentially cut into 6 μm sections and mounted onto slides. Slides were deparaffinized in xylene and hydrated with gradient ethanol. Antigen retrieval was performed with 0.1% trypsin. The slides were blocked in 1% BSA and 1:50 diluted normal donkey serum. Normal rabbit IgG was used as negative control, and Snai1, E-cadherin, and Notch2 antibodies with various dilutions were added to the slides, detected with Alexa-488 labeled donkey anti-rabbit antibody and, imaged by a Leica TCS SP5 confocal system.

Immunohistochemistry for ferritin was performed on a NEXes automated stainer (Ventana Medical Systems, Tucson AZ). Heat antigen retrieval was performed in 0.01M citrate. Rabbit anti-mouse ferritin (Abcam, Cambridge MA) was diluted 1:500, detected with biotinylated goat anti-rabbit (Vector Laboratories Burlingame, CA) and, visualized with streptavidin-HRP and diaminobenzidene. Slides were counterstained with hematoxylin. Snail was studied similarly with a 1:200 dilution.

### Iron therapy assay

Thirty 3-week-old Balb/c mice were randomly divided into three groups. One group (10 mice) was fed 350 ppm iron and the other two groups were fed 3.5 ppm iron. After 11 weeks, each mouse from one group of iron deficient mice received an intraperitoneal (*i*.*p*.) injection of 0.5 mg iron dextran (100 mg/ml Fe, Sigma-Aldrich, St. Louis, MO), which is a clinically relevant dose equivalent to a human dose of 1.0 g per 60 kg, [18] since a mouse weighs about 30 g. The diet for this group was then switched to 350 ppm iron diet. One week later, all mice were inoculated with 5 × 10^4^ 4T1 cells into mammary fat pads. Tumor volumes were measured and tissue samples were collected and processed.

### Association of hemoglobin with lymph node status in young BC patients

Through the Department of Pathology at the Fourth Military Medical School in Xi’an, China, there were a total of 148 BC patients < 45 years old diagnosed between year 2008 and year 2010 (Additional file 2: Table S2). Because ferritin and transferrin saturation were not available, hemoglobin (Hb) levels were used to compare between lymph node positive and negative groups. Patients’ personal information was recorded in such a manner that subjects cannot be identified directly or through identifiers linked to the subjects. This study is exempted from the Institutional Review Board (IRB) by the Fourth Military Medical University. Age, hemoglobin levels, and lymph node status at the time of diagnosis were used.

### Statistical Analyses

Statistical evaluations of the mouse data were conducted by Student's t-test for paired comparison or by one-way analysis of variance for multiple comparisons, followed by a post hoc Newmann-Keuls test. p-value < 0.05 was considered to be significantly different. For human data, Wilcoxon test was used to compare Hb levels between node positive and node negative groups, and boxplots were used to demonstrate the distributional difference between the two groups. Logistic regression with node positivity as response and Hb levels and age as predictors was used to assess association of Hb levels with lymph node positivity.

## Results

### Effects of iron deficiency on tumor growth and metastasis

Previous studies have shown that, despite a 10-fold spread in iron contents from each diet, mice fed 35 ppm and 350 ppm iron have normal iron status and those fed 3.5 ppm iron are iron deficient [14,19]. Here mild iron deficiency was observed in Balb/c mice fed 3.5 ppm iron as reflected by a transferrin saturation of < 20% and lowered serum iron. Mice fed 35 ppm and 350 ppm iron had adequate iron status (Additional file 3: Figure S1).

**Figure 1:**
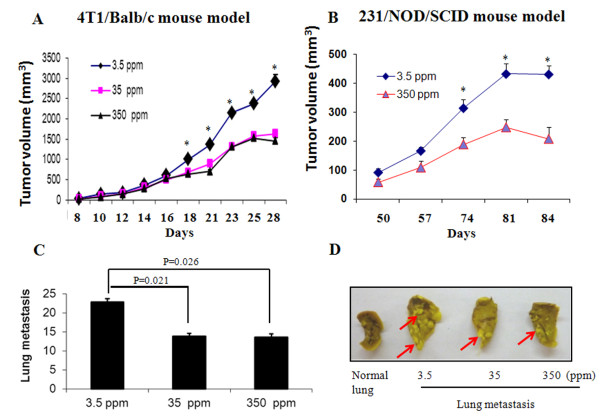
**Host iron deficiency promotes mouse mammary tumor and human BC growth and metastasis. (A)** Balb/c mice were fed standard rodent diets containing 3.5 ppm Fe (iron deficient), 35 ppm Fe (normal low), and 350 ppm Fe (normal high), respectively. After 12 weeks, 5 × 10^4^ mouse 4T1 mammary cancer cells were subcutaneously (*s*.*c*.) injected into the flanks of the mice. **(B)** NOD/SCID mice were fed 3.5 ppm and 350 ppm Fe diets. After 12 weeks, 1 × 10^6^ human BC MDA-MB-231 cells were *s*.*c*. injected into mammary fat pad. **(C)** Average number of tumor nodules per lobe of the lung in each diet group from the 4T1/Balb/c mouse model. **(D)** Representative metastatic lungs from each diet group of the 4T1/Balb/c mouse model. Arrow indicates tumor nodules. *: Significantly different from the group of mice fed 350 ppm Fe diet.

Subsequently, these mice were inoculated with 4T1 cells into the right flank. From day 18, primary tumor volumes were significantly higher in iron deficient mice compared to the two groups of mice fed normal iron diets (2922.6 ± 161.8 mm^3^ in 3.5 ppm Fe *vs* 1636.5 ± 86.6 mm^3^ in 35 ppm Fe, or 1453.8 ± 90.6 mm^3^ in 350 ppm Fe, mean ± SEM, p < 0.05, n=9) (Figure 1A).

To investigate whether this observation is true with human BC cells, NOD/SCID mice were fed 3.5 and 350 ppm iron for 12 weeks, followed by *s*.*c*. injection of 1 × 10^6^ human BC MDA-MB-231 cells into the mammary fat pads. Figure 1B shows that primary tumor volumes from these human cancer cells were also significantly higher in iron deficient mice. Because of injection of tumor cells into the mammary fat pads, tumor volumes from MDA-MB-231 cells were smaller than those from 4T1 cells injected into the flanks of the mice.

In addition, surface lung metastases were significantly greater in the iron deficient Balb/c mice, with approximately 22.9 ± 2.6 nodules per lobule in 3.5 ppm Fe *vs* 13.9 ± 2.4 in 35 ppm Fe, or 13.7 ± 2.7 in 350 ppm Fe (p < 0.05, n=9, Figure 1C). Bousin’s staining demonstrated that the entire lung was significantly invaded by 4T1 tumors in iron deficient mice and fewer tumor metastasis to the lung were observed in mice fed normal iron diets (Figure 1D). These results indicate that iron deficiency promotes growth and metastasis of BC of both mouse and human origins.

### Effects of iron deficiency on epithelial mesenchymal transition (EMT)

**Figure 2:**
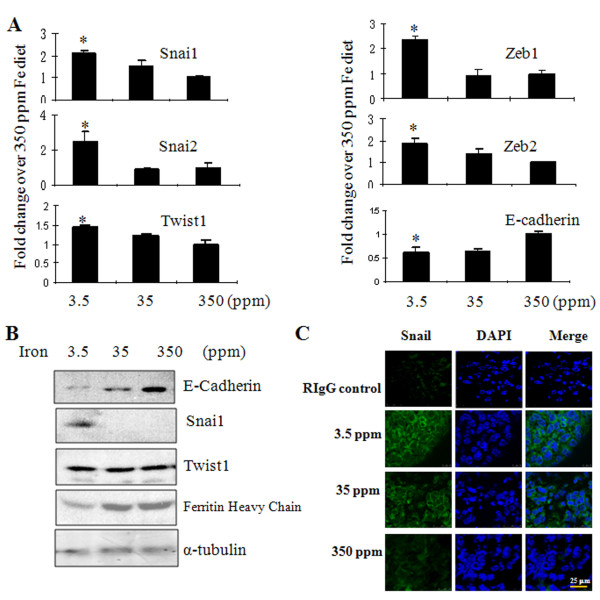
**Host iron deficiency activates EMT pathway. (A)** The mRNA expression profiles for EMT markers were normalized to the geomean of three housekeeping genes, GAPDH, G6PD, and HPRT1 and expressed as fold changes over the control mice fed 350 ppm Fe diet. **(B)** Alteration of E-cadherin, Snai1, Twist1, and ferritin proteins in primary tumors of mice fed iron-deficient diet. α-tubulin was used as a loading control. **(C)** Increased expression of Snai1 in primary tumors of mice fed iron-deficient diet by immunofluorescence. Tumor tissues were stained with normal rabbit IgG (control) or Snai1 antibody (Green) and DPAI (Blue). *: Significantly different from the group of mice fed 350 ppm Fe diet.

To explore molecular mechanisms by which iron deficiency potentiates metastasis, we searched EMT markers [20]. EMT is characterized by decreases in epithelial markers, such as E-cadherin, which is driven by transcription repressors, such as Snail, Twist and Zeb [21]. We found that these events occurred in tumor-bearing iron deficient mice, as mRNA levels of Snai1, 2 and Zeb1, 2 were upregulated at least one-fold in the primary tumors of mice fed an iron deficient diet (Figure 2A). Consistent with qPCR results, protein levels of E-cadherin were dramatically decreased but levels of Snai1 were greatly augmented by iron deficiency (Figure 2B). Ferritin, an iron storage protein, was low in the primary tumors from mice fed iron deficient diet. Immunofluorescence showed an iron dose-dependent decrease in Snai1 expression (Figure 2C).

### Effects of iron deficiency on Notch, TGF-β, and WNT signaling

**Figure 3:**
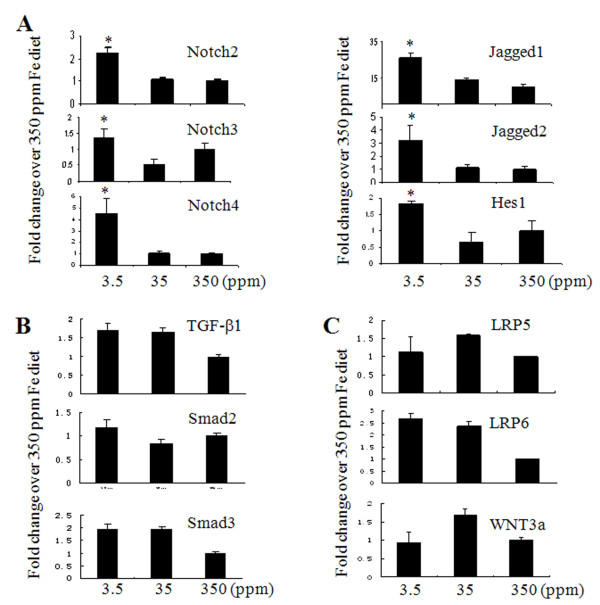
**Host iron deficiency stimulates Notch but not TGF-β, and Wnt signaling. (A)** Iron deficiency increased mRNA expression of Notch 2, 3, 4, their receptors Jagged 1, 2 and Hes1 by qPCR. **(B)** No significant changes for TGF-β signaling pathway components, TGF-β1, Smad 2, and Smad3. **(C)** No significant changes for Wnt signaling pathway components, LRP5, LRP6, and Wnt3a. *: Significantly different from the group of mice fed 350 ppm Fe diet.

To determine which pathway is responsible for iron deficiency-mediated EMT, we examined changes in transforming growth factor-beta (TGF-β), wingless-int (WNT), and Notch signaling [22, 23]. We found that mRNA levels of Notch 2, 3, and 4 and their ligands Jagged1, 2 and Hes1 were increased approximately 1–4 folds in primary tumors from iron deficient mice (Figure 3A). There were no significant differences in mRNA levels of TGF-β and WNT signaling pathways among the three diet groups (Figure 3B and 3C). These results suggest that host iron deficiency mainly alters Notch signaling, leading to EMT activation.

### Effects of iron supplementation on EMT and tumor growth and metastasis

**Figure 4:**
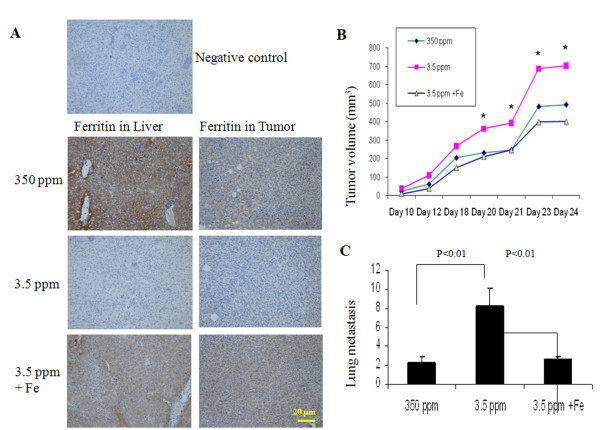
**Iron therapy improves iron deficiency and decreases tumor growth and metastasis. (A)** Immunohistochemistry of ferritin showed that iron therapy replenished iron in liver and corrected iron-deficient status in Balb/c mice fed iron-deficient diet, magnification x20; **(B)** Iron therapy inhibited tumor growth and **(C)** lung metastasis.

To show that iron deficiency is responsible for the observed tumor growth and metastasis, we reversed iron deficiency by injecting iron dextran one week before tumor xenograft. Using immunohistochemistry (IHC), we found that iron dextran replenished iron levels. Striking differences in liver ferritin exist between iron-deficient mice and iron-deficient mice receiving iron treatment or mice fed normal iron diet (Figure 4A; left column). Differences in ferritin levels in the primary tumors between iron-deficient mice and mice fed normal iron or receiving iron supplement were not as striking (Figure 4A). We also found that iron deficient mice have higher tumor volume than mice fed normal iron diet (925.6 ± 147.4 mm^3^*vs*. 644.8 ± 53.2, p < 0.05, n=9). Most importantly, iron therapy significantly inhibited iron deficiency-related tumor growth (638.8 ± 90.3 *vs*. 925.6 ± 147.4 p < 0.05, n=9) (Figure 4B). While iron deficiency enhanced lung metastasis, iron therapy decreases lung metastasis (Figure 4C). In this experiment, instead of injecting 4T1 cells into the flanks (Figure 1A), they were injected into the mammary fat pads, resulting in smaller tumor volumes and less numbers of lung metastasis.

**Figure 5:**
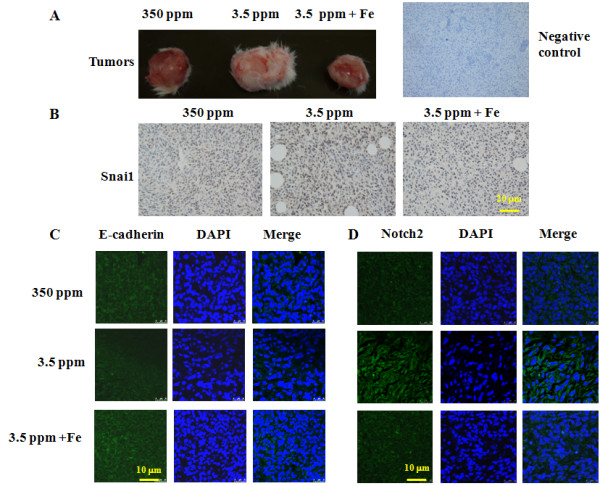
**Iron therapy reverses iron deficiency-mediated EMT markers. (A)** Representative primary tumors from each group; **(B)** Iron therapy downregulates Snai1 to the levels in mice fed normal 350 ppm iron diet by immunohistochemistry; **(C)** Iron therapy upregulates E-cadherin and **(D)** reduces Notch2 when compared to mice fed iron deficient diet by immunofluorescence.

Not only did iron therapy reverse lung metastasis, but also reversed iron deficiency-mediated tumor growth (Figure 5A), downregulated Snai1 (Figure 5B), upregulated E-cadherin (Figure 5C), and decreased Notch2 expression (Figure 5D). These results strongly indicate that iron deficiency leads to tumor growth and metastasis.

### Association of hemoglobin (Hb) with lymph node invasion in young BC patients

**Figure 6:**
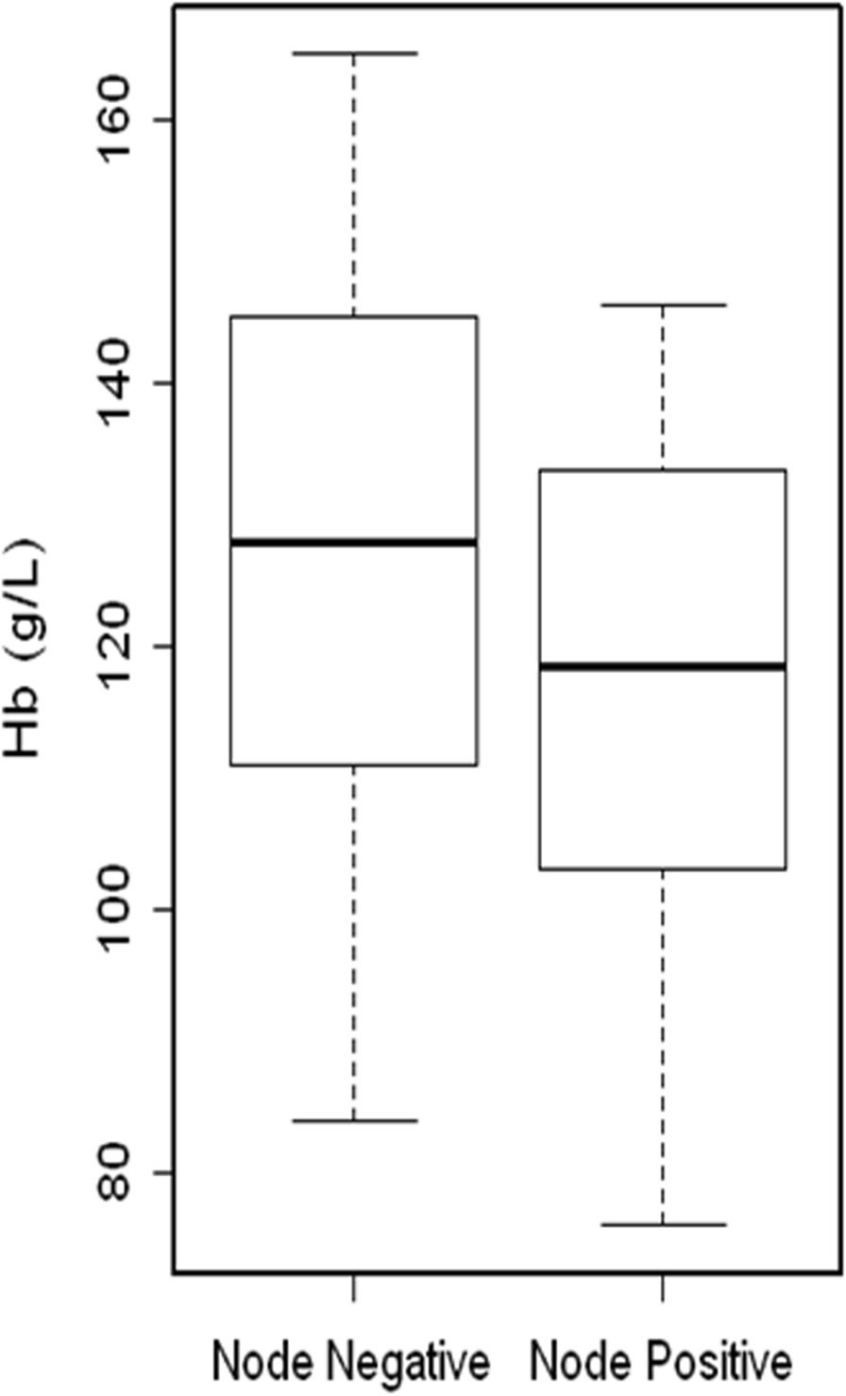
**Association of hemoglobin (Hb) levels with lymph node status in young BC patients.** The cohort included 62 subjects with lymph node invasion (mean age 34.5 years) and 74 subjects without lymph node invasion (mean age 35.4 years). Mean levels of Hb in patients with lymph node invasion were 119.6 g/l and those without lymph node invasion were 126.7 g/l (p < 0.002).

To show that the above observation is clinically relevant, Hb levels were used to compare between node positive and node negative patients. We found that Hb level is significantly lower (p<0.002) in node positive BC patients (n=62, mean age: 34.5 years old, mean Hb ± SD: 119.6 ± 14.5 g/l, 95% CI: 116, 123.2) than node negative BC patients (n=74, mean age: 35.5 years old, mean Hb ± SD: 126.7 ± 13.6 g/l, 95% CI: 123.6, 129.8) (Figure 6).

## Discussions

Iron is long known to be a double-edged sword. Previous studies have shown that iron-accumulation-associated pro-oxidant conditions in cancer cells could fuel the activity of iron-dependent proteins and enable enhanced cancer cell proliferation, contributing to both tumor initiation and tumor growth [12]. In the present study, we provide evidence that host iron deficiency-mediated proangiogenic environment could lead to xenografted tumor growth and metastasis as well. Although our study is limited to BC, which has the most epidemiological data on recurrence in young patients, it confirms previous observation that recurrence of other cancers, such as colorectal cancer, tends to have a higher incidence of recurrence in young patients [24]. By linking young age to iron deficiency and then to BC malignancy, our finding may have identified that host iron deficiency represents a clinical entity that is specific to young women and is associated with lower survival and higher recurrence in young BC patients. In support of our view, microarray data from two age-specific cohorts (young, ≤ 45 years, and older, ≥ 65 years) found major gene sets unique to breast tumors in young women. These include the mTOR/rapamycin/hypoxia pathway and cancer angiogenesis and metastasis [1]. In contrast, no common gene sets were found among tumors arising in older women. By comparing the gene expression profile from young BC patients to those from mice fed iron deficient diet, [1,13] overlaps of genes centered on hypoxia, angiogenesis, and metastasis. This supports our finding that a link may exist between host iron deficiency and BC malignancy [25].

Moreover, “functional” iron deficiency known as anemia of chronic disease (ACD) is a clinical condition where stored iron is sufficient but circulating iron is deficient. ACD is a well-established risk factor for increased morbidity and mortality of late stage cancer patients [26-28]. It develops as cancer progresses or when cancer patients undergo chemotherapy. “Absolute” iron deficiency refers to the depletion of iron stores in the body and is highly prevalent in young women as results of menstruation and insufficient dietary iron uptake [8,9]. If ACD increases mortality in cancer patients, “absolute” iron deficiency could have the same effects. Indeed, our animal data showed that mice fed 3.5 ppm iron had a transferrin saturation of < 20%, [19] characterizing a mild iron deficiency. This moderate iron deficiency significantly affected tumor growth and metastasis in both immuno-competent and immuno-deficient mice. Furthermore, human studies suggest that mild iron deficiency without advancing to anemia could be a cause of lymph node invasion, because Hb levels in the node positive BC patients were only slightly below normal (119.6 g/L compared to cutoff value of 120 g/L) and iron deficiency proceeds anemia in young women.

EMT is critical in cancer metastasis, which involves physical translocation of a cancer cell to a distant organ and the ability of the cancer cell to develop a metastatic lesion at the distant site [20]. EMT pushes cancer cells to undergo changes from epithelial to mesenchymal phenotype. We found that primary tumors grown in iron deficient mice were characterized by decreased expression in E-cadherin and increased expression in Snai1. In addition, Notch, but not TGF-β or WNT pathway, was affected, suggesting that iron deficiency-mediated Notch signaling is a likely driving force for this event. In view of the importance of iron deficiency in hypoxia inducible factor-1alpha (HIF-1α) stabilization and the role of HIF-1α in Notch signaling, [29,30] iron deficiency-mediated HIF-1α is a relevant pathway to be investigated in the future.

To further confirm that iron deficiency is a causative factor of BC growth and metastasis, we corrected iron deficiency by injecting iron dextran before inoculation of cancer cells in mice. Iron supplementation replenished iron in the liver. Most importantly, it not only reduced tumor growth and lung metastasis, but also reversed iron deficiency-mediated upregulation of Snai1 and Notch and downregulation of E-cadherin.

Iron supplementation is a standard therapy to correct iron deficiency. However, the suitability of iron therapy for young BC patients needs further investigation. Our study is designed to prove that iron deficiency leads to BC aggressiveness and, thus, we used a protocol to correct iron deficiency before inoculation of cancer cells. It remains unknown how cancer cells would respond to iron treatment when iron is administrated after cancer cell inoculation. To indicate the utility of iron supplementation during chemotherapy, it has been shown that amenorrhea (no menstruation) for at least six months significantly increases overall survival in young BC patients, regardless of chemotherapy and estrogen receptor status [31]. The average blood loss during menstruation is 35 mL per month [32] and every 100 mL blood contains about 50 mg of iron [33]. We estimate that iron loss for a period of 6 months is in the amount of 105 mg (35 mL/month × 6 months × 50 mg/100mL). We wonder whether 105 mg iron supplementation during chemotherapy could increase the overall survival in young BC patients with menses. As of now, in clinical practice in oncology, evaluation of iron deficiency is usually not required during BC diagnosis and, thus, not treated during BC therapy. Although clinical trial with iron supplementation in young BC patients requires a further high level of preclinical evidence, this research adds one more reason for practitioners to check whether iron levels in young BC patients are up to their FDA-recommended levels.

## Conclusions

In the present study, we showed that iron deficiency, highly prevalent in young women because of the menstruation, could contribute to poor prognosis in young BC patients. By linking young age to iron deficiency and then to BC malignancy, our study may have identified iron deficiency as a clinically treatable risk factor for young BC patients.

## Abbreviations

EMT: Epithelial mesenchymal transition; ESA: Erythropoietin-stimulating agents; Hb: Hemoglobin; Her2: Human epidermal growth factor receptor 2; HIF: Hypoxia inducible factor; IDA: Iron deficiency anemia; PR: Progesterone receptor; TGF-β: Transforming growth factor-β; WNT: Wingless-int.

## Competing interest

The authors declare that there are no conflicts of interest pertaining to the contents of this article.

## Authors’ contributions

JJ and QY carried out the mouse xenograft experiments, characterized protein and gene expression profile of the tumors, and summarized the animal data. YS performed the statistical analysis of the human data. DA, JS, and BS participated in the design of the study and helped to draft the manuscript. SK and LC carried out the immunohistochemistry and histological assays. ZY and JL retrieved human breast cancer data from the hospitals. XH conceived of the study, participated in its design and coordination, and drafted the manuscript. All authors read and approved the final manuscript.

## Pre-publication history

The pre-publication history for this paper can be accessed here:

http://www.biomedcentral.com/1471-2407/13/307/prepub

## Supplementary Material

Additional file 1:Table S1. The primer sequences of the oligonucleotides used for qPCR. Click here for file

Additional file 2: Table S2. Hemoglobin and lymph nodes status in human breast cancer patients. Click here for file

Additional file 3: Figure S1. Body iron status in mice fed three different levels of iron diets. Serum iron and transferrin saturation (TS) rate in mice fed 3.5 ppm iron diet (iron deficient), 35 ppm and 350 ppm iron diets (normal low and normal high iron levels. Click here for file
