# Supplementary material for: A link between premenopausal iron deficiency and breast cancer malignancy
Source: BMC Cancer. 2013 Jun 24;13:307. doi: 10.1186/1471-2407-13-307 (PMC3716572; doi:10.1186/1471-2407-13-307)
Supplement: Additional file 1:Table S1. — The primer sequences of the oligonucleotides used for qPCR. [file 1471-2407-13-307-S1.pdf]

Online Table 1: The primer sequences of the oligonucleotides used for qPCR,

**EMT Markers**	Forward 5'-	Reverse 5'
m Snai1	CTTGTGTCTGCACGACCTGT	GGAGCAGGAGAATGGCTTC
m Snai2	ACAGCGAACTGGACACACAC	CGAGGTGAGGATCTCTGGTT
m ZEB1	GCATGTGACCTGTGTGACAA	CACACTCGTGAGGCCTCTTA
m ZEB2	CCTTCTGCGACATAAATACGAA	CGAGTGCTCGATAAGGTGGT
m TWIST1	CGGACAAGCTGAGCAAGATT	GCAGGACCTGGTACAGGAAG
m TWIST2	ACCAGTGAGGAAGAGCTGGA	TCGCTCGATTTCTTGCTGTA
m E-cadherin	CCTGCCAATCCTGATGAAAT	CGAACACCAACAGAGAGTCG
vimentin	TGGTTGACACCCACTCAAAA	GGTCATCGTGATGCTGAGAA
**Notch pathway**		
m Notch2	GAATGGGGCCAACAGAGATA	TTTGGCCGCTTCATAACTTC
m Notch3	AAAAATGGAGCCAACAAGGA	CAGCTTGGCAGCCTCATAG
m Notch4	AGGCTGGAGCGGATAAAGAT	AGGAAAAGCGGCGTCTGT
m HES1	TCTGGAAATGACTGTGAAGCA	CGGTATTTCCCCAACACG
m Jagged2	TTGTTATGGGTGGCTCTTCC	AGCCACAGCACACTGAACAC
m Jagged1	GAGGCGTCCTCTGAAAAACA	GAGCTCAGCAGAGGAACCAG
VEGF A	CCTGCAAAAACACAGACTCG	CGCCTTGGCTTGTCACAT
Hif-2 alpha	CTCCAGGAGCTCAAAAGGTG	CAGTTCCGGCAACAGGTAAG
Hif-1 alpha	ATTCTCCAAGCCCTCCAAGT	GGTCTGCTGGAACCCAGTAA
**WNT pathway**		
Lrp5	GCCACAGACCCCTCTCTCTA	GCCATACCTCGAATGACGTAG
Lrp6	TCCAACAGTCCTTCCACACA	AAAGTGCCGGTAGCTGTACG
SOST(Sclerostin)	CCTCCTCCTGAGAACAACCA	GCAGCTGTACTCGGACACAT
DKK1	GGGGATGGATATCCCAGAAG	GTCTGATGATCGGAGGCAGA
DKK2	TAGGAAGGCCACACTCCAAG	GTAGGCATGGGTCTCCTTCA
DKK3	ACTGGAGCCTGAAGGAGCTT	GGCTTGCACATGTACACCAG
sfrp-1	TCAGAGGCCATCATTGAACA	TGGGGACAATCTTCTTGTCA
sfrp-2	CGACATCATGGAAACCCTTT	CAGGATGATCTTGGTGTCTCTG
sfrp-4	TGAAAAGTGGAGAGATCAACTCAG	CTGCTGTTCCTGAAGCCTCT
mWnt3a	CCATGAACCGTCACAACAAT	CTTGAGGTGCATGTGACTGG
mWnt5a	AACAATGAAGCAGGCCGTAG	GAGCCAGACACTCCATGACA
Wnt4	CCGGGCACTCATGAATCTT	CGCATGTGTGTCAAGATGG
Wnt7b	CGCCTCATGAACCTTCACA	CCTGACACACCGTGACACTT
Wnt10b	AGAATGCGGATCCACAACA	CACTTCCGCTTCAGGTTTTC
Wif1	AACCCAACAAGTGCCAGTGT	ATGAGGCTGGCTCCATACC
mCyclinD1	CACAACGCACTTTCTTTCCA	TCCAGAAGGGCTTCAATCTG
