# Supplementary material for: A link between premenopausal iron deficiency and breast cancer malignancy
Source: BMC Cancer. 2013 Jun 24;13:307. doi: 10.1186/1471-2407-13-307 (PMC3716572; doi:10.1186/1471-2407-13-307)
Supplement: Additional file 2: Table S2. — Hemoglobin and lymph nodes status in human breast cancer patients. [file 1471-2407-13-307-S2.pdf]

Online Table 2: Hemoglobin and lymph nodes status in human breast cancer patients

Age	No. of Cases	No. of Lymph Node Positive	No. of Lymph Node Negative	No. of Unknown	% of Total Patients
20-30	10	7	3	0	6.8
31-40	78	32	44	2	52.7
41-45	60	23	35	2	40.5
Total	148*	62	82	4	100

* In our 148 subject cohort, 8 subjects with ductal carcinoma *in situ* (DCIS) in the lymph node negative group and 4 subjects with unknown status were removed. The cohort included 62 subjects with lymph node invasion (mean age 34.5 years) and 74 subjects without lymph node invasion (mean age 35.4 years). Patients are all Chinese.
