# Supplementary material for: A link between premenopausal iron deficiency and breast cancer malignancy
Source: BMC Cancer. 2013 Jun 24;13:307. doi: 10.1186/1471-2407-13-307 (PMC3716572; doi:10.1186/1471-2407-13-307)
Supplement: Additional file 3: Figure S1. — Body iron status in mice fed three different levels of iron diets. Serum iron and transferrin saturation (TS) rate in mice fed 3.5 ppm iron diet (iron deficient), 35 ppm and 350 ppm iron diets (normal low and normal high iron levels. [file 1471-2407-13-307-S3.pptx]

## Slide 1
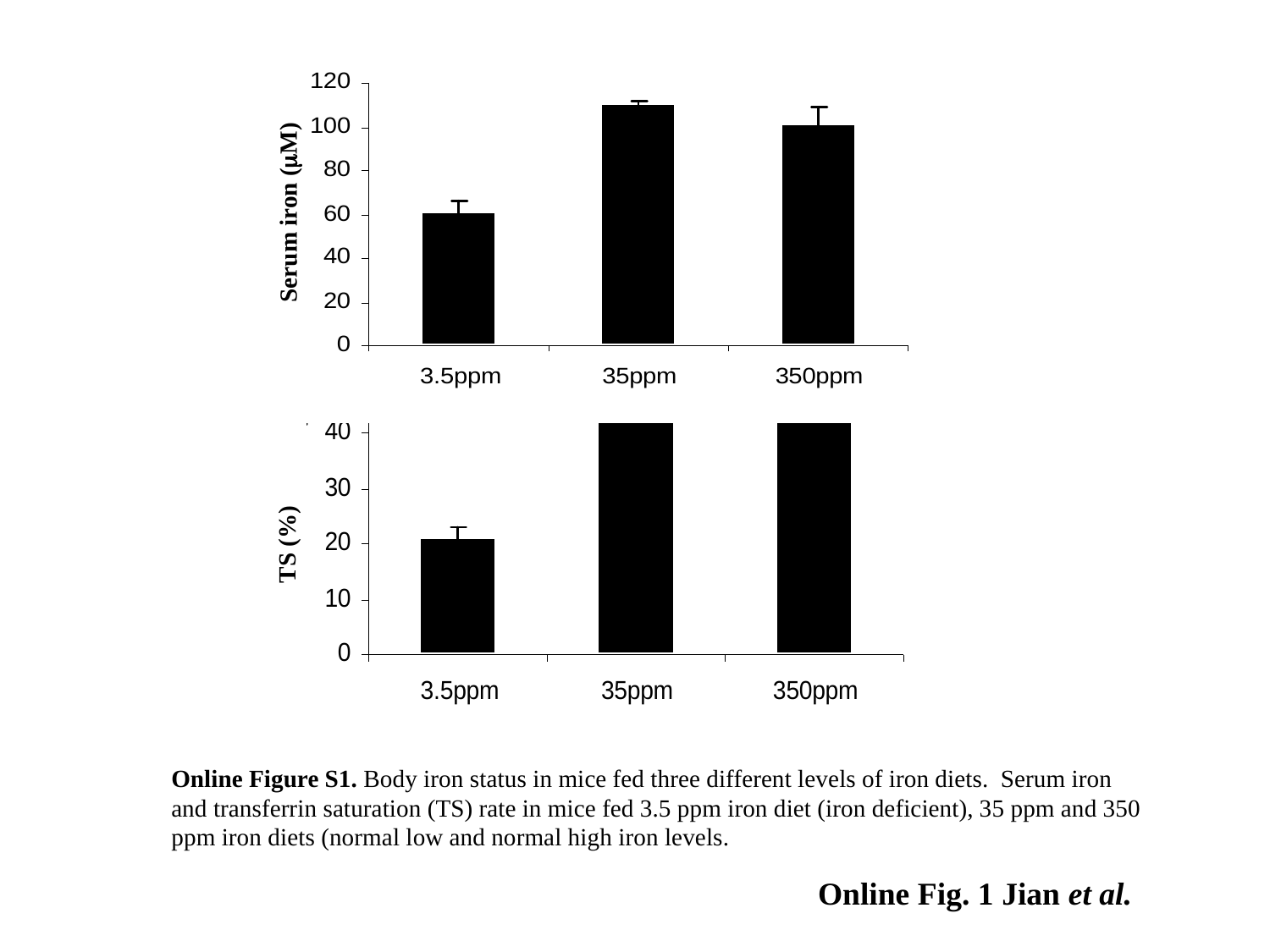

Serum iron (mM)
TS (%)
Online Figure S1. Body iron status in mice fed three different levels of iron diets. Serum iron and transferrin saturation (TS) rate in mice fed 3.5 ppm iron diet (iron deficient), 35 ppm and 350 ppm iron diets (normal low and normal high iron levels.
Online Fig. 1 Jian et al.
